# Incidence of Dementia in Canada: A National Trend Analysis of Newly Diagnosed Cases

**DOI:** 10.7759/cureus.90418

**Published:** 2025-08-18

**Authors:** Oluwatomiwa S Fasoro, Faith C Anyanwu, Ajibola O Jayeola, Okelue E Okobi, Joan O Osaigbovo, Angela C Onojedje, Rukayat O Balogun, Oluchi C Abah, Aimuamwosa Okoro, Chinasa Okeke-Chikelu

**Affiliations:** 1 Clinical Sciences, College of Health Sciences at Obafemi Awolowo University, Ile Ife, NGA; 2 Community Health and Primary Health Care, Lagos State University Teaching Hospital, Lagos, NGA; 3 General Practice, Ladoke Akintola University of Technology, Ogbomosho, NGA; 4 Family Medicine, Larkin Community Hospital Palm Springs Campus, Miami, USA; 5 Family Medicine, Nnamdi Azikiwe University Teaching Hospital, Nnewi, NGA; 6 General Medicine, Madonna University, Ogene, NGA; 7 Family Medicine, South Tyneside and Sunderland NHS Foundation Trust, Sunderland, GBR; 8 Family and Community Medicine, Abia State University, Uturu, NGA; 9 General Medicine, I. Horbachevsky Ternopil National Medical University, Ternopil, UKR; 10 Faculty of Medicine, Windsor University School of Medicine, Basseterre, KNA

**Keywords:** aging population, alzheimer's disease, canada, dementia, epidemiology, healthcare planning, incidence trends, regional disparities

## Abstract

Introduction: Dementia is a progressive neurocognitive disorder affecting aging populations worldwide. Understanding its incidence trends is essential for planning preventive strategies and healthcare services, particularly in countries with aging demographics like Canada. This study aimed to examine national trends in the age-standardized incidence rates of newly diagnosed dementia, including Alzheimer's disease, among Canadians aged 65 years or older from 2007 to 2022.

Methods: We conducted a retrospective, population-based trend analysis using the Canadian Chronic Disease Surveillance System (CCDSS), administered by the Public Health Agency of Canada (PHAC). The CCDSS aggregates de-identified administrative health data supplied by provincial and territorial health ministries, including physician billing claims, hospital discharge abstracts, and health insurance registry data. Dementia cases were ascertained using validated International Classification of Diseases (ICD)-9/ICD-10 diagnostic codes for Alzheimer’s disease and related dementias applied to physician and hospital records (case definition and code list per CCDSS protocols). The study period covered fiscal years 2007-2008 through 2021-2022, with supplementary data for 2022-2023 where available. Age-standardized incidence rates were calculated using the 2011 Canadian standard population. Data completeness for physician billing and hospital discharge records exceeded 95% in participating jurisdictions; however, some provinces/territories had partial or missing submissions in certain years. All analyses used aggregated, de-identified counts provided by CCDSS; no individual-level records were accessed.

Results: In 2022, the crude incidence rate of newly diagnosed dementia among Canadians aged 65 years and older was 1,323 per 100,000 population per year (95%CI: 1315-1331). By sex, females had a higher crude incidence (1,437 per 100,000 population per year; 95%CI: 1425-1449) than males (1,194 per 100,000 population per year; 95%CI: 1183-1206). Incidence increased markedly with advancing age: 610 per 100,000 population per year (95%CI: 604-617) in those aged 65-79, and 3,669 per 100,000 population per year (95%CI: 3640-3697) among individuals aged more than 80. Regional disparities were observed: Nunavut had the highest age-standardized rate (1,700 per 100,000 population per year; 95%CI: 921-2969) while Saskatchewan had the lowest (1,154 per 100,000 population per year; 95%CI: 1108-1203). From 2007 to 2022, the age-standardized incidence declined overall; this pattern may potentially reflect improvements in prevention, risk-factor control, or early detection, although causal attribution cannot be established from these data alone.

Conclusion: Although the age-standardized incidence of newly diagnosed dementia in Canada declined modestly between 2007 and 2022, substantial sex, age, and regional disparities remain. The findings emphasize the need for ongoing investment in dementia prevention, equitable diagnostic access, and region-specific interventions. With the aging population, coordinated public health strategies remain essential to sustain progress and reduce the future burden of dementia on individuals, caregivers, and healthcare systems.

## Introduction

Dementia is a progressive neurological disorder that gradually impairs memory, thinking, behavior, and the ability to carry out daily activities [1]. It significantly affects the quality of life of individuals and their families, often requiring long-term care and support. As the condition advances, it can lead to complete dependency and severe cognitive decline. While there is currently no cure, early diagnosis and management can help improve outcomes and delay progression [2,3]. As life expectancy rises globally, the number of older adults at risk of developing dementia is growing rapidly, leading to increased social, economic, and healthcare challenges [4]. In Canada, dementia not only impacts individuals and families but also places a significant strain on long-term care systems and health services. Early identification and monitoring of incidence trends are essential to guide effective prevention and resource allocation strategies at the national level [5].

As of 2021, 57 million people live with dementia globally, with nearly 10 million new cases annually. Alzheimer’s disease accounts for 60-70% of cases. Dementia is the seventh leading cause of death and a major cause of disability in older adults. In 2019, it cost the global economy US$1.3 trillion, half from informal caregiving. Women are disproportionately affected, facing higher mortality and providing 70% of care hours [6]. As of January 1, 2025, an estimated 771,939 people in Canada were living with dementia, with over 414 new cases diagnosed daily. By 2030, nearly one million Canadians could have dementia, a 65% increase from 2020, alongside 187,000 new cases annually (a 51% rise). In the 2040s, over 20,000 people could be diagnosed each month. By 2050, more than 1.7 million Canadians may be living with dementia, with 685 new cases daily. Between 2020 and 2050, 6.3 million Canadians will develop, live with, or die from dementia [7]. 

Dementia refers to a group of neurodegenerative disorders, including Alzheimer's disease, vascular dementia, Lewy body dementia, and frontotemporal dementia, all marked by progressive and irreversible cognitive decline [1,6,8]. Though the underlying mechanisms vary, they commonly involve neuronal loss, synaptic dysfunction, and brain atrophy, leading to impairments in memory, language, executive function, and behavior. In Alzheimer’s disease, the most prevalent form, beta-amyloid plaques and neurofibrillary tangles of hyperphosphorylated tau disrupt neuronal communication, beginning in the hippocampus [9,10]. Vascular dementia arises from reduced cerebral blood flow due to strokes or small vessel disease, while Lewy body dementia involves abnormal alpha-synuclein protein aggregates. Contributing processes such as oxidative stress, neuroinflammation, and mitochondrial dysfunction are shared across types [11]. As damage progresses, widespread atrophies occur in regions like the temporal and parietal lobes. Pathology varies, with Alzheimer's linked to amyloid and tau, and vascular or mixed dementias associated with infarcts [12]. Risk factors include age, genetics (e.g., APOE-e4), and modifiable ones like hypertension, diabetes, obesity, smoking, and physical inactivity, while protective factors include education, mental stimulation, and a healthy diet [9-12].

This study uses the Canadian Chronic Disease Surveillance System (CCDSS), a national surveillance platform maintained by the Public Health Agency of Canada (PHAC), which compiles de-identified administrative health data from provincial and territorial jurisdictions (physician billing claims, hospital discharge abstracts, and health insurance registries). For dementia ascertainment, the CCDSS applies a validated case-finding algorithm that identifies individuals as having dementia if they meet any of the following criteria: (i) one or more hospital separation records with a dementia/Alzheimer's disease diagnosis, (ii) three or more physician claims for dementia separated by at least 30 days within a two-year interval, or (iii) one or more prescriptions for cholinesterase inhibitor medications used to treat Alzheimer's disease. The diagnostic codes used to capture dementia events in hospital and physician records follow standard International Classification of Diseases (ICD) classifications (ICD-9 codes for historical billing data, such as 290.x and 331.x, and ICD-10 codes including F00-F03, G30, and related codes). When the CCDSS dementia algorithm has been validated against family physician electronic medical records, it demonstrated good specificity and moderate sensitivity (reported sensitivity ≈ 79.3% and specificity ≈ 99.1% in older adults), supporting its suitability for population-level surveillance while acknowledging known under-ascertainment of milder or undiagnosed cases [13,14]. 

The primary objective of this study is to evaluate national trends in the age-standardized incidence of newly diagnosed dementia cases among Canadians aged 65 years and older between 2007 and 2022. The secondary objective is to compare crude incidence rates by sex and age group.

## Materials and methods

Data source and study design

This study employed a retrospective, population-based design using data from the CCDSS, a national surveillance platform administered by the PHAC [14]. The CCDSS aggregates de-identified administrative health data supplied by provincial and territorial health ministries, including health insurance registries, physician billing claims, and hospital discharge abstracts, and applies standardized case definitions to produce national and jurisdictional estimates of chronic disease incidence and prevalence. Detailed CCDSS methods and the full, jurisdiction-level case definitions are available from PHAC. The current analysis spans fiscal years 2007-2008 through 2021-2022, with supplementary data available for 2022-2023 where jurisdictions provided it. 

Study participants and questionnaires

The study population included all individuals aged 65 years and older who were eligible for provincial or territorial health insurance coverage during the study period. Dementia cases (including Alzheimer’s disease and related dementias) were identified using the CCDSS dementia case definition and diagnostic code lists applied to hospital records, physician billing claims, and prescription data. According to CCDSS case-ascertainment rules used across jurisdictions, a person was classified as having dementia if they met any of the following criteria: (i) at least one hospital separation record with a dementia or Alzheimer’s disease diagnosis code, (ii) at least three physician claims for dementia, each separated by at least 30 days within a two-year period, or (iii) at least one prescription record for a cholinesterase inhibitor. These criteria are documented in PHAC publications and provincial CCDSS reports.

The diagnostic codes used follow standard ICD classifications. Commonly applied categories include ICD-9 codes such as 290 (senile and presenile organic psychotic conditions) and 331 (other cerebral degenerations, including Alzheimer’s disease), and ICD-10 codes such as F00-F03 (dementia syndromes) and G30 (Alzheimer’s disease). Jurisdictions may use additional related codes depending on available coding fields, but the definitions are standardized to ensure comparability across provinces and territories.

No patient questionnaires were administered, as case ascertainment relied exclusively on administrative health data. Individuals who already met CCDSS dementia criteria in the historical data before the 2007-2008 baseline were excluded from incident case counts so that analyses reflected only newly ascertained cases during the study period.

Data collection and quality assurance

Data were collected systematically by provincial and territorial health ministries and transmitted to PHAC following national CCDSS protocols. Each jurisdiction applied a standardized algorithm to identify dementia cases, ensuring comparability. Data quality was maintained through regular audits, cross-validation against health records, and adherence to inclusion/exclusion criteria defined by the CCDSS. The source database provides only aggregated frequency (percentage) data, without access to raw individual-level counts; therefore, all results are reported as percentages where applicable. Records were de-identified before analysis to protect patient privacy. The completeness of physician billing and hospital discharge data was over 95%, supporting reliable national estimates.

Variables of interest

The primary outcome variable was the age-standardized incidence rate of newly diagnosed dementia per 100,000 persons aged 65 years or older, calculated annually. Secondary variables included crude incidence rates stratified by sex (male/female) and age groups (65-74, 75-84, and ≥85 years) for the fiscal year 2022-2023. Additional demographic information, such as province of residence, was used for national aggregation, though regional analyses were not the primary focus. Dementia types (e.g., Alzheimer's disease, vascular dementia) were not distinguished due to coding limitations.

Data analysis and statistical methods

Age-standardized incidence rates were computed using the 2011 Canadian standard population. The annual percent change (APC) and average annual percent change (AAPC) were estimated with 95% confidence intervals (CIs). For 2022-2023 crude incidence comparisons, statistical significance between subgroups was assessed using non-overlapping 95% CIs.

Ethical considerations

This study used aggregated, de-identified data from the CCDSS, which did not involve direct patient contact or access to individual-level records. As per Canadian guidelines, additional institutional research ethics board approval was not required for secondary analyses of de-identified surveillance data. All data handling adhered to the Tri-Council Policy Statement on Ethical Conduct for Research Involving Humans, relevant provincial privacy legislation, and established data-sharing agreements with participating health authorities. CCDSS data are distributed under the Open Government Licence, Canada, and were securely managed to ensure confidentiality throughout the study [14].

## Results

This section presents findings on the incidence of newly diagnosed dementia cases, including Alzheimer's disease, in Canada among individuals aged 65 years and older. Results are organized under national trends over time, differences by region, and an international comparison of dementia prevalence in 2021.

Overall incidence of dementia in 2022

In 2022, the crude incidence rate of newly diagnosed dementia cases, including Alzheimer's disease, among Canadians aged 65 years and older was 1,323 per 100,000 population (95%CI: 1315-1331). This national figure reflects the burden of new dementia diagnoses within the aging population across all provinces and territories.

Incidence of dementia based on sex (2022)

**Figure 1:**
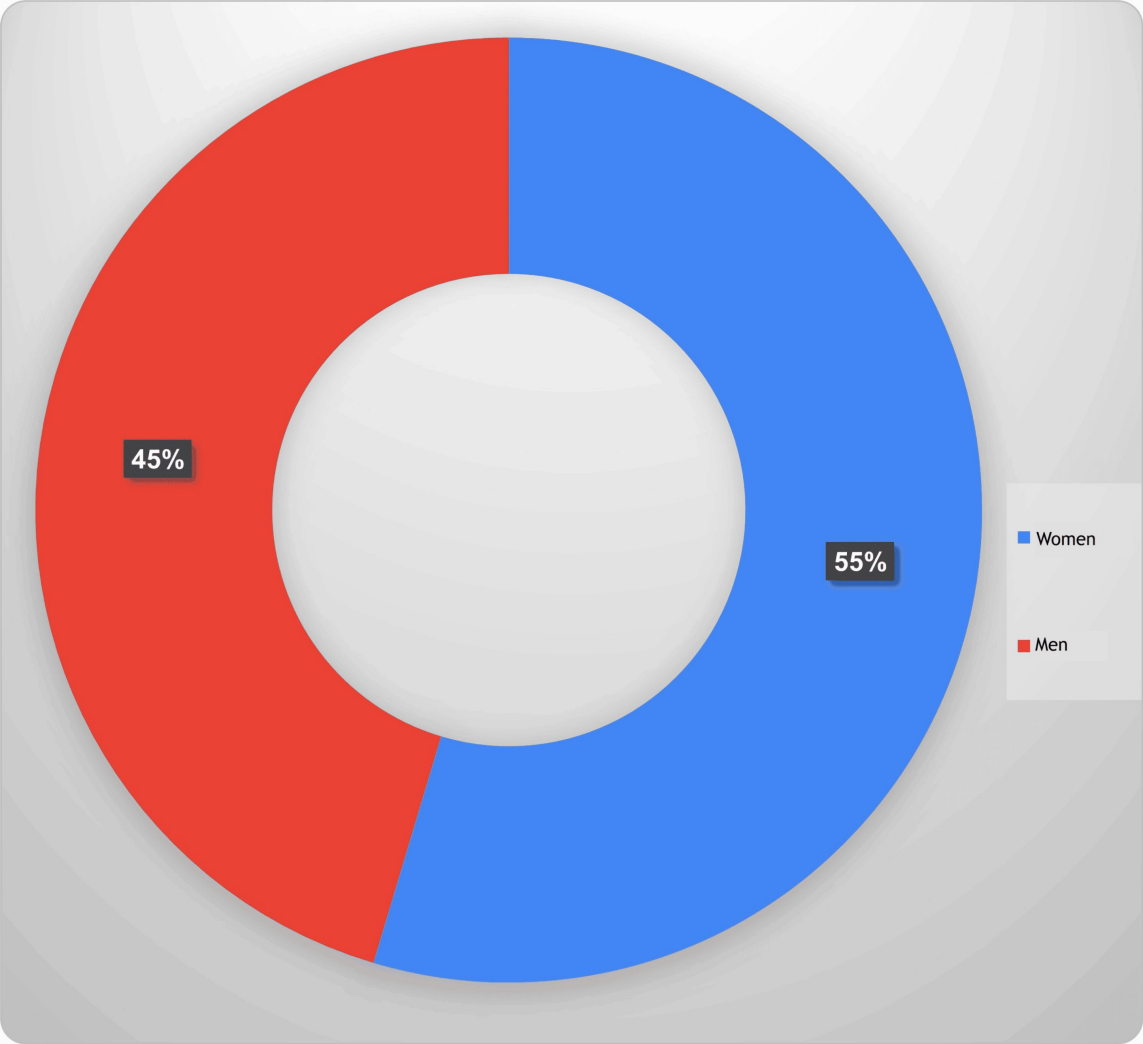
Sex-based differences in the incidence of newly diagnosed dementia cases in Canada, 2022

When analyzed by sex, women had a higher incidence rate of newly diagnosed dementia compared to men. The crude incidence rate for women was 1,437 per 100,000 (95%CI: 1425-1449), while the rate for men was 1,194 per 100,000 (95%CI: 1183-1206). The non-overlapping 95%CIs indicate a statistically significant difference, suggesting that women in this age group are more likely to be diagnosed with dementia than men. Figure 1 presents the sex-based differences in the incidence of newly diagnosed dementia cases in Canada. 

Incidence of dementia based on age group (2022)

A steep increase in dementia incidence was observed with advancing age. Among seniors aged 65-79 years, the crude incidence rate was 610 per 100,000 (95%CI: 604-617). In contrast, individuals aged 80 years and older experienced a markedly higher incidence rate of 3,669 per 100,000 (95%CI: 3,640-3,697). The large disparity between the two age groups, coupled with non-overlapping confidence intervals, confirms that age remains a significant risk factor for developing dementia.

National trends in dementia incidence (2007-2022)

**Figure 2:**
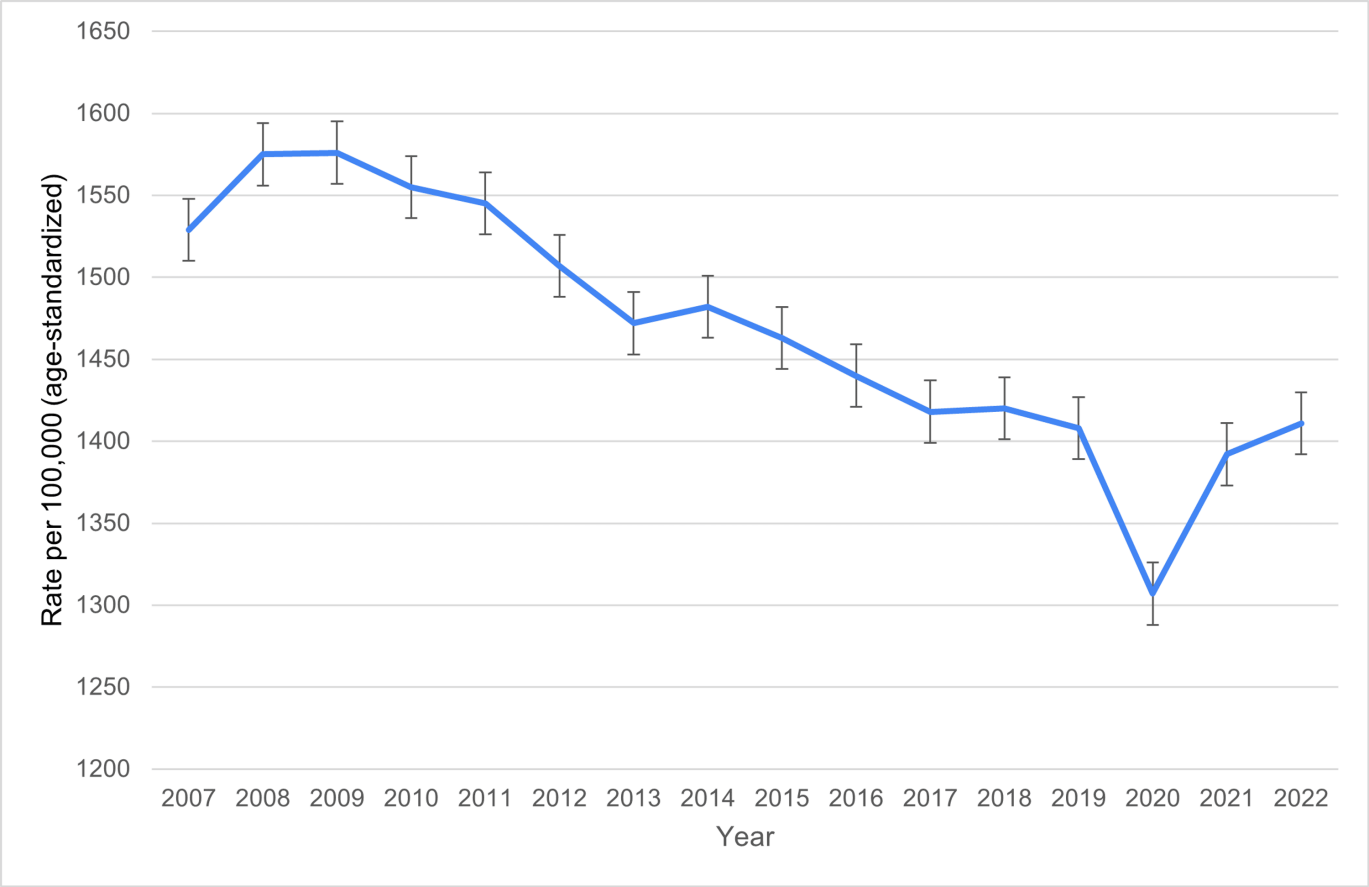
National trends in age-standardized dementia incidence in Canada (2007–2022)

Over the 15-year period from 2007 to 2022, Canada witnessed a gradual decline in the age-standardized incidence rate of newly diagnosed dementia cases among older adults. In 2007, the incidence rate was 1,529 per 100,000 population, which rose slightly in 2008 (1,575) and 2009 (1,576). However, from 2009 onward, a consistent downward trend was observed. By 2019, the rate had dropped to 1,408 per 100,000, followed by a more substantial decline in 2020 to 1,307 per 100,000. The 2020 dip may reflect, at least in part, reduced healthcare contact and delays in diagnostic assessment during the COVID-19 pandemic; this is consistent with national surveillance reports and public health summaries that documented declines in health service utilization and temporary drops in recorded chronic disease diagnoses during the early pandemic period in Canada [14]. In 2021 and 2022, incidence rates rebounded modestly to 1,392 and 1,411 per 100,000, respectively. Figure 2 shows that age-standardized dementia incidence rates declined gradually in Canada from 2007 to 2022, with a notable dip during the COVID-19 period.

Despite this slight increase, the overall pattern indicates a notable long-term decrease in new dementia diagnoses, suggesting the potential impact of improved health literacy, better management of risk factors (e.g., hypertension, diabetes), and enhanced public health awareness around cognitive health in aging.

Regional variation in dementia incidence (2022)

In 2022, age-standardized incidence rates of newly diagnosed dementia cases varied notably across Canadian provinces and territories, highlighting important regional disparities. The highest rate was recorded in Nunavut, at 1,700 per 100,000 population (95%CI: 921-2969). However, this figure should be interpreted with caution due to the wide confidence interval reflecting a small and less stable population base. Table 1 presents the age-standardized incidence rates of newly diagnosed dementia cases, including Alzheimer's disease, among Canadians aged 65 and older across provinces and territories in 2022.

**Table 1:** Regional age-standardized incidence rates of newly diagnosed dementia cases per 100,000 population aged 65 years and older, Canada, 2022 NA: Not available

Region	Rate per 100,000 (95% CI )
British Columbia	1388 (1365 – 1411)
Alberta	1423 (1394 – 1453)
Saskatchewan	1154 (1108 – 1203)
Manitoba	1445 (1396 – 1496)
Ontario	1407 (1393 – 1421)
Quebec	1456 (1437 – 1474)
New Brunswick	N/A
Nova Scotia	1485 (1434 – 1538)
Prince Edward Island	1294 (1170 – 1428)
Newfoundland and Labrador	1288 (1221 – 1359)
Yukon	1168 (864 – 1555)
Northwest Territories	N/A
Nunavut	1700 (921 – 2969)

Among the provinces, Nova Scotia had the highest incidence at 1,485 per 100,000 (95%CI: 1434-1538), followed closely by Quebec (1,456) and Manitoba (1,445). On the other end of the spectrum, Saskatchewan reported the lowest incidence rate at 1,154 per 100,000 (95%CI: 1108-1203), while Newfoundland and Labrador (1,288) and Prince Edward Island (1,294) also fell below the national average. In contrast, more populous provinces such as Ontario and British Columbia reported incidence rates that aligned closely with the national average, 1,407 per 100,000 (95%CI: 1393-1421) and 1,388 per 100,000 (95%CI: 1365-1411), respectively. Some jurisdictions, including New Brunswick and the Northwest Territories, did not submit data for 2022. These regional differences may be influenced by a range of factors, such as differences in population aging, healthcare access, diagnostic practices, public awareness, and broader social determinants of health.

International comparison of dementia prevalence (2021)

**Figure 3:**
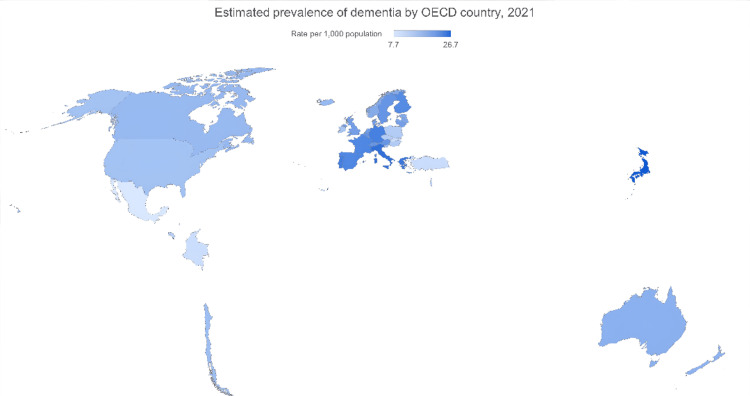
International comparison of dementia prevalence per 1,000 population (2021) Image Credit: Authors; Data Source: Organisation for Economic Co-operation and Development (OECD) dementia statistics [15], World Health Organization dementia fact sheet [6], and the Alzheimer’s Disease Facts and Figures report [16]

Figure 3 presents the international comparison of dementia prevalence per 1,000 population in 2021. Countries are compared based on reported prevalence estimates among older adults. From an international perspective, Canada’s dementia prevalence in 2021 was 13.9 per 1,000 population, placing it below the Organization for Economic Co-operation and Development (OECD) average of 15.7 [13]. Compared to countries such as Japan (26.7) and Italy (23.7), Canada’s rate was significantly lower, indicating more favorable conditions in terms of dementia burden. It ranked similarly to Australia (15.1) and New Zealand (14.7), and slightly ahead of the United States (12.7) and Ireland (12.2), based on prevalence estimates reported by the Organisation for Economic Co-operation and Development (OECD) [15], the World Health Organization (WHO) [6], and Alzheimer’s Disease Facts and Figures [16]. These relatively favorable outcomes may reflect Canada’s success in implementing preventive health strategies, promoting early diagnosis, and ensuring equitable access to healthcare services. Nonetheless, given the country’s rapidly aging population, continued surveillance and proactive interventions remain critical to managing future dementia trends. Canada’s dementia prevalence in 2021 was below the OECD average, with lower rates than Japan and Italy but similar to Australia [13].

## Discussion

Dementia is a progressive neurological syndrome that profoundly affects individuals, families, healthcare systems, and larger societies. This national trend analysis of newly diagnosed dementia cases in Canada from 2007 to 2022 explores how age-standardized and crude incidence rates have changed over time and vary by age, sex, and geography. Findings show a gradual decline in age-standardized incidence yet persistent disparities, highlighting the need for tailored responses. This discussion integrates clinical practice, molecular pathology, public health strategy, and policy frameworks to contextualize the results and guide future action.

Over 15 years, the age-standardized incidence of dementia among Canadians aged 65 or older decreased from 1,529 to 1,411 per 100,000. A marked drop during the COVID-19 pandemic, reaching 1,307 per 100,000 in 2020, likely reflects reduced access to diagnosis. This interpretation aligns with Canadian evidence showing a decline in dementia-related physician visits and diagnostic assessments during the pandemic, as documented in national health service utilization reports [13,14]. PHAC data also indicate disruptions in non-urgent neurological evaluations during early 2020, particularly in primary care and memory clinics [13,14]. These disruptions mirror patterns observed internationally but provide a Canadian-specific basis for the observed decline. Despite this dip, long-term trends match international findings. For example, the Framingham Heart Study observed a roughly 20% per decade decline in dementia incidence, attributed to better cardiovascular care and education [17]. This parallel suggests that Canadian health efforts are having a meaningful impact.

Canada’s public health landscape has emphasized societal risk factors since the Lancet Commission’s report, which stated that up to 40% of dementia cases are attributable to modifiable risks such as hypertension, diabetes, smoking, depression, and inactivity [18,19]. National initiatives, including blood pressure and smoking cessation programs, nutrition interventions, and social engagement campaigns, are likely contributing factors behind the decline in incidence, especially in urban and suburban regions with robust healthcare infrastructure.

Nevertheless, the absolute burden of dementia remains high. In 2022, 1,323 cases per 100,000 Canadians aged 65+ were documented, illustrating the continued importance of vigilance and response. Disparities by sex and age are especially notable. Women experienced a higher crude incidence (1,437 per 100,000) compared to men (1,194 per 100,000), with non-overlapping confidence intervals indicating statistical significance [13]. Life expectancy explains part of this difference, yet emerging research indicates biological, hormonal, and immunological factors also contribute. Estrogen withdrawal after menopause may reduce neural resilience, while women are more prone to autoimmune disorders and depression, both associated with greater dementia risk [20,21].

Age likewise remains a central determinant. In 2022, individuals aged 80 and above had an incidence of 3,669 per 100,000 compared to 610 per 100,000 for those aged 65-79. Neuropathology confirms that aging correlates with greater accumulation of amyloid plaques, tau tangles, and cerebrovascular burden, often presenting as mixed pathology [22]. Studies show that even delaying symptom onset by a couple of years can significantly reduce future prevalence [23]. This underscores the need for lifelong prevention and risk reduction efforts.

Dementia is not a single entity but a syndrome driven by neuropathological mechanisms. Alzheimer's disease, the most common subtype, is defined by amyloid-β plaque deposition and hyperphosphorylated tau tangles, leading to synaptic failure and neuronal death predominantly in the hippocampus [24]. In vascular dementia, chronic hypoperfusion and infarctions impair cortical and subcortical function. Lewy body dementia involves α-synuclein aggregation [25]. Shared pathways across subtypes include mitochondrial dysfunction, oxidative stress, and chronic neuroinflammation [26].

Genetic risk factors, notably the APOE ε4 allele, contribute significantly, especially in Alzheimer's disease and particularly among women [27]. For example, Canadian clinical guidelines highlight that dementia subtypes often converge in practice. A typical patient may present with both Alzheimer-type changes, such as hippocampal atrophy associated with APOE ε4 status, alongside vascular lesions on MRI, thereby meeting criteria for mixed Alzheimer-vascular dementia [28]. Such multimodal diagnostic approaches highlight the necessity of comprehensive evaluation in clinical practice.

Geographically, the highest age-standardized incidence in 2022 was observed in Nunavut at 1,700 per 100,000, though confidence intervals were wide. Among provinces, Nova Scotia (1,485), Quebec (1,456), and Manitoba (1,445) reported elevated rates, while Saskatchewan (1,154) and Prince Edward Island (1,294) were below the national mean [13]. Factors contributing to this variability may include population age structure, service availability, lifestyle factors, and socioeconomic inequities. Indigenous communities and northern populations often face higher comorbid vascular disease rates and limited care access, and previous studies link these to greater dementia prevalence [29]. To address these concerns, culturally sensitive screening, equitable resource allocation, and training for local caregivers are imperative. Because they are from culturally and linguistically diverse backgrounds, barriers such as language, sociocultural factors, isolation, low awareness of dementia and stigma, their environment, including inaccessibility of transport and healthcare services, impact on their cultural inclusivity, and overall health outcomes [30]. To address these concerns, culturally sensitive screening, equitable resource allocation, and training for local caregivers are imperative. Specific and tailored dementia-friendly initiatives and communities have been proposed, leveraging existing cultural leaders and social structures, while addressing identified barriers [30]. Also, following the World Health Organization's recognition of traditional healers as community stakeholders in dementia care and prevention, a pathway to culturally safe care is documented. However, due to their underrepresentation and marginalization in healthcare systems, due mostly to the lack of culturally safe dementia care (CSDC) policies at community and national levels globally, a call to action is underway for policy improvements and eventual implementation of the WHO Global Action Plan on the Public Health Response to Dementia (2017-2025) [31].

Comparatively, Canada’s dementia prevalence in 2021 stood at 13.9 per 1,000 population, below the OECD average of 15.7. Countries like Japan (26.7) and Italy (23.7) had higher rates, possibly due to older demographics [15]. Canada’s lower prevalence may reflect its universal healthcare system, public health emphasis, and dementia-focused strategies like the National Dementia Strategy (2019) and Canadian Consortium on Neurodegeneration in Aging [28]. However, demographic forecasts predict that dementia cases in Canada will rise substantially, potentially exceeding 1.7 million by 2050 [13].

Parallel trends are observed in the United States, where prevalence remains high but incidence is declining, attributed to improved lifestyle and cardiovascular risk management [16]. Both the National Healthy Brain Initiative in the United States and Canada’s dementia support frameworks, including the National Dementia Strategy and the Fifth Canadian Consensus Conference on the Diagnosis and Treatment of Dementia (CCCDTD5), highlight the importance of prevention alongside medical and social support services [5,28,32].

Funding and planning remain essential, given the projected care demands. In 2019, Canada spent over CAD 10 billion on dementia care, much of it non-medical and driven by informal caregiving [33]. Informal caregivers, predominantly women, bear a considerable emotional and financial burden. Their decline in well-being can indirectly heighten their own risk of cognitive decline, underscoring the need for resources, respite, and mental health support [34].

Diagnosis and treatment protocols guided by the CCCDTD5 emphasize early detection through tools like the Montreal Cognitive Assessment (MoCA), MRI, and inclusive specialist involvement [28]. Pharmacological options, cholinesterase inhibitors and memantine, offer symptom relief but do not prevent progression [35]. Emerging therapies such as monoclonal anti-amyloid agents (lecanemab) are under review in Canada; however, although promising, they present challenges in cost and equitable delivery [36].

Non-pharmacological interventions, such as cognitive stimulation, physical exercise, social engagement, and occupational therapy, are strongly supported by evidence and are particularly valuable when initiated early [37]. Broader social determinants, including education, poverty, housing, and food access, need attention if incidence and outcomes are to further improve [19,37].

The essential takeaway is twofold. First, Canada’s efforts to reduce dementia incidence appear promising, especially in urban areas with strong public health infrastructures. Second, the rise in absolute case numbers, demographic and geographic disparities, and the limitations of current treatments demand sustained, multifaceted solutions, clinically, scientifically, and socially.

To address the growing burden of dementia, Canada must implement a multifaceted and inclusive national response. Expanding equitable access to prevention and diagnostic services across the country, particularly in underserved and rural communities, is essential to ensure early detection and timely intervention. Efforts should also focus on intensifying midlife and older-adult risk reduction through comprehensive chronic disease management, the promotion of healthy lifestyle behaviors, and enhanced mental health care. Strengthening support systems for informal caregivers who bear the emotional and physical weight of dementia care is critical to reducing caregiver burnout and its associated health consequences. Furthermore, continued investment in molecular and clinical research, including large-scale clinical trials and the development of accurate and accessible biomarkers, will play a vital role in advancing early diagnosis and treatment. Finally, to achieve meaningful and equitable progress, surveillance and prevention programs must fully integrate social determinants of health, addressing factors such as education, income, housing, and social inclusion across diverse population groups.

Recent advancements in diagnostic tools have significantly improved the early detection of Alzheimer’s disease, enabling intervention before significant cognitive decline. Techniques such as positron emission tomography (PET), particularly amyloid and tau PET, allow direct visualization of pathological protein deposits in the brain, even during the preclinical phase. Cerebrospinal fluid (CSF) biomarkers like the Aβ42/40 ratio, total tau, and phosphorylated tau remain gold standards for diagnosis, while newer technologies such as single molecule array (SIMOA) and mass spectrometry have enhanced biomarker detection with remarkable sensitivity [22,24,28]. Blood-based biomarkers, including p-tau181, p-tau217, and neurofilament light chain (NfL), are emerging as cost-effective, scalable alternatives that correlate strongly with disease progression and imaging findings [24,16].

Diagnostic innovation has also expanded into non-traditional and digital domains. MRI can detect early atrophy in memory-related regions such as the hippocampus and entorhinal cortex. Retinal imaging via optical coherence tomography (OCT) and hyperspectral imaging offers a non-invasive method to mirror cerebral pathology [38,39]. Meanwhile, digital biomarkers and artificial intelligence-based tools now detect subtle behavioral or cognitive changes through passive monitoring using wearables, speech patterns, or smartphone tests. These multimodal strategies reflect a shift toward early, accessible, and proactive Alzheimer’s detection in real-world settings.

With dementia incidence increasing sharply among those aged 80 and above, it is critical to consider the compounding effect of geriatric syndromes such as frailty, multimorbidity, and polypharmacy. Frailty heightens susceptibility to cognitive decline, while multiple chronic conditions (e.g., diabetes, cardiovascular disease) contribute to neurodegeneration through inflammatory and vascular pathways [6,18,26]. Concurrent polypharmacy may further exacerbate risk by introducing drug interactions and medication-induced cognitive impairment [3]. Together, these interrelated factors form a cascading vulnerability, increasing the likelihood of progression to dementia, particularly in the oldest old [2,4].

Globally, multimodal, community-driven prevention strategies are gaining traction. The Finnish Geriatric Intervention Study to Prevent Cognitive Impairment and Disability (FINGER) demonstrated that integrating nutritional guidance, physical activity, cognitive training, and vascular risk control led to a 25% improvement in global cognition compared to controls [40]. This success catalyzed the formation of the World-Wide-FINGERS consortium, which has adapted the model across over 25 countries, including Canada’s own Canadian Therapeutic Platform Trial for Multidomain Interventions to Prevent Dementia (CAN-THUMBS-UP) initiative [41]. Evidence from these randomized trials shows benefit even in high-risk groups such as APOE ε4 carriers and individuals with vascular comorbidities.

Canada can further strengthen its national response by embedding such culturally tailored, FINGER-style interventions into primary care and senior services. Leveraging existing public health infrastructure to deliver group-based lifestyle counseling, cognitive training, physical activity, and risk monitoring could significantly delay cognitive decline, reduce incidence, and improve equity in brain health across regions [42].

Strengths and limitations

One of the primary strengths of this study is its national scope, utilizing comprehensive administrative health data to capture longitudinal trends in dementia incidence across 15 years (2007-2022). The inclusion of age-standardized and crude rates, along with subgroup analyses by sex, age, and province, enhances the reliability and comparability of the findings. This granularity allows for detailed regional assessments, which are vital for informing health policy at both provincial and federal levels. Additionally, the use of standardized diagnostic coding practices strengthens the consistency of case identification across Canada’s health systems. However, the study is not without limitations. First, reliance on administrative data may lead to underdiagnosis or misclassification, particularly in early or mild cases of dementia that do not come to clinical attention. Second, the dataset lacks clinical details, such as dementia subtypes (e.g., Alzheimer's disease vs. vascular dementia), severity, and comorbidities. Third, data from certain territories and provinces (e.g., New Brunswick, Northwest Territories) were missing in some years, potentially affecting national estimates. Additionally, social and behavioral risk factors (e.g., education, lifestyle, income) could not be assessed, limiting the interpretation of the underlying causes behind regional disparities. Lastly, the COVID-19 pandemic may have temporarily disrupted dementia diagnoses due to reduced healthcare access, possibly skewing rates in 2020-2021. Despite these limitations, the study provides an essential epidemiological foundation for understanding and responding to Canada’s growing dementia burden.

## Conclusions

This national trend analysis offers valuable insight into the evolving epidemiology of dementia in Canada, particularly among individuals aged 65 years and older. While the age-standardized incidence rate of newly diagnosed dementia cases has declined modestly from 2007 to 2022, the disease remains a significant public health burden, especially among women, the oldest age group (≥80 years), and certain provinces such as Nova Scotia and Quebec. Part of this modest decline may be attributable to Canadian public health initiatives that have promoted cardiovascular health, diabetes prevention, and cognitive well-being, such as the Canadian Dementia Strategy and regional campaigns targeting blood pressure control, physical activity, and hearing loss awareness. These programs, along with chronic disease management integration in primary care and expanded access to early screening, have likely contributed to risk reduction, though disparities in incidence highlight the persistent gaps in equitable healthcare access and awareness across provinces.

With Canada’s aging population projected to grow sharply in the coming decades, sustaining and amplifying this downward trend will require multi-pronged, well-resourced efforts. Examples include national and provincial public health campaigns that raise awareness of modifiable dementia risk factors such as hypertension, obesity, and hearing loss through culturally tailored outreach; the integration of dementia risk reduction into routine chronic disease management in primary care; and the development of age-friendly community initiatives that encourage social participation, physical activity, and safe living environments for older adults. Equally important will be the expansion of specialized services, including memory clinics and geriatric care, particularly in rural and underserved areas where diagnostic delays and care shortages remain common. Overall, the findings of this study reinforce the need for sustained, coordinated, and cross-sectoral action to reduce dementia risk, enhance timely diagnosis, and improve outcomes for older Canadians regardless of geographic location or demographic group.
